# All-inside meniscal repair surgery: factors affecting the outcome

**DOI:** 10.1007/s10195-015-0342-2

**Published:** 2015-02-21

**Authors:** Haroon Majeed, SaravanaVail Karuppiah, Kohila Vani Sigamoney, Guido Geutjens, Robert G. Straw

**Affiliations:** 1 University Hospital of North Staffordshire, Stoke-on-Trent, ST4 6QG UK; 2 Trauma and Orthopaedics, Royal Derby Hospital, Derby, UK; 3 Royal Derby Hospital, Derby, UK

**Keywords:** Meniscal preservation, Meniscal anchors, Knee meniscus, Knee arthroscopy, Failure of meniscal repair

## Abstract

**Background:**

Meniscal injury is currently a well-recognized source of knee dysfunction. While it would be ideal to repair all meniscus tears, the failure rate is significantly high, although it may be reduced by careful selection of the patients. Our objective was to assess the outcome of meniscal repair surgery and the role of simultaneous reconstruction of the anterior cruciate ligament (ACL).

**Materials and methods:**

Retrospectively, all consecutive patients between January 2008 and 2011 who underwent meniscal repair were included. Patients were identified using the hospital database with diagnosis and procedure codes. Patient notes were reviewed, including details of the type of tear, chronicity, location, and surgery. We used symptomatic resolution as the outcome measure.

**Results:**

136 Meniscal repairs were performed in 122 patients with a mean age of 26.8 years. Mean follow-up duration was 9 months. 63 % of the patients underwent medial and 37 % underwent lateral meniscal repair, with failure rates of 19 % for medial and 12 % for lateral menisci. Ligament injuries were found in 61 % of the patients (*n* = 83). Failure of meniscal repair occurred in 14.5 % (*n* = 12) of the patients who had early ACL reconstruction and in 27 % (*n* = 22) of the patients who had delayed ACL reconstruction (*p* = 0.0006). The failure rate was found to be 13 % in patients who were younger than 25 years (61 %) and 15 % in patients who were older than 25 years (39 %).

**Conclusion:**

The success rate of meniscal repair was found to be significantly better when ACL reconstruction was performed simultaneously with meniscal repair.

**Level of evidence:**

Level IV.

## Introduction

Meniscal injury is currently a well-recognized source of knee dysfunction, and its arthroscopic treatment has become one of the most commonly performed orthopedic procedures around the world [1]. Meniscal resection is usually performed more commonly than repair, but there has been a shift in focus from meniscal resection to meniscal preservation and repair in recent years [1]. The meniscus withstands different forces, including shear, tension, and compression, and plays a crucial role in load-bearing, load transmission, and shock absorption. The contact area of a tibiofemoral joint surface may decrease by up to 20 % following a partial meniscectomy and by 50–70 % following a total meniscectomy. Hence, the resultant increase in contact stresses accelerates the progression of degenerative arthritis following a meniscectomy [2]. The development of arthritis following meniscal resection surgery may take up to 10–15 years in the case of a medial meniscus, but it may happen within 2 years in the case of a lateral meniscus [3].

**Fig. 1:**
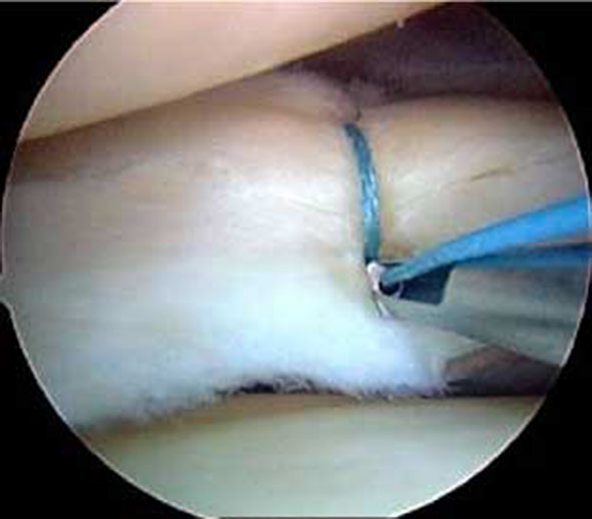
Meniscal repair using the vertical mattress technique

**Fig. 2:**
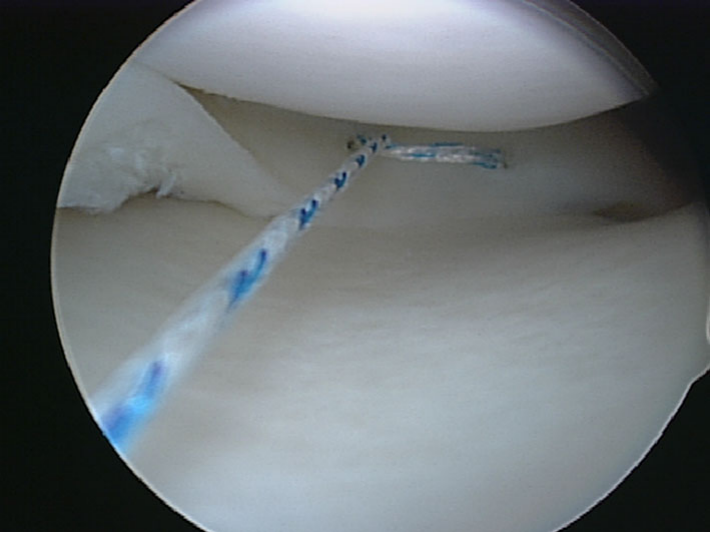
Meniscal repair using the horizontal mattress technique

The techniques employed for meniscal repair have also evolved in recent years. First-generation techniques for meniscal repair were based on Henning’s technique (first inside-out repair, 1980) [4], but the potential risk of neurovascular damage has been a major concern for this type of repair. Russell Warren [5] introduced an outside-in technique which aimed at reducing neurovascular complications. In recent years, an all-inside technique has been introduced, which is widely used currently (Figs. 1, 2) [6].

It would be ideal to repair all meniscus injuries; however, the failure rate has been found to be significantly high and the implant costs considerable, requiring careful consideration and selection of the patients. Some studies have reported success rates for meniscal repair to be up to 60–90 % depending on the region of meniscal repair [7–10]. Meniscal repairs performed in conjunction with ACL reconstruction are generally thought to have a better healing rate than meniscal repair in knees with intact ACLs [7]. The objective of our study was to assess the outcome of meniscal repair surgery, focusing in particular on meniscal healing when the surgery is performed in conjunction with ACL reconstruction.

## Materials and methods

We performed a retrospective review covering 3 years (January 2008–2011) in a large teaching hospital. All consecutive patients who underwent meniscal repair were included. The data were collected through patients’ case notes and included demographic details, mechanism of injury, symptoms and their durations, details of the meniscal tear (type, location, size, age), postoperative rehabilitation regimen, concurrent surgical procedure, recurrent symptoms, and subsequent surgeries performed. We analyzed the data from different perspectives, comparing the outcomes for medial and lateral menisci, tears of different ages (more than 6 weeks and less than 6 weeks old), different age groups of patients (over 25 and under 25 years); menisci repaired with and without ACL reconstruction, and repairs in different zones of menisci.

All of our patients underwent arthroscopic meniscal repair performed using FasT-Fix anchors (Smith & Nephew^®^). The all-inside technique was used in all our patients. Postoperatively, the range of knee flexion was limited from 0 to 90° using an off-the-shelf knee brace for 6–8 weeks in order to protect the repaired menisci, followed by gradual rehabilitation with physiotherapy. Weight-bearing was allowed as tolerated, except in those patients who had multiligament reconstruction. This regime was followed in all our patients after surgery. We used symptomatic resolution (pain, swelling, and locking) as the outcome measure in order to assess the success rate. Statistical analysis was done using Fisher’s exact test (two-tailed) for categorical variables and Student’s *t* test (two-tailed) for numerical variables.

## Results

One hundred thirty-six meniscal repairs were performed in 122 patients during our study period. The male to female ratio was 4:1. Age ranged from 11 to 58 years (mean 26.8 years). In male patients, age ranged from 11 to 49 years (mean 25 years), and in female patients it ranged from 20 to 58 years (mean 35.6 years). Mean follow-up duration was 9 months (1–26 months). The emergency department was the main source of referrals (53 %), followed by primary care (25 %) and the physiotherapy department (14 %). Main symptoms included pain (94 %), swelling (68 %), and mechanical locking (38 %). Instability (47 %) was seen predominantly in patients with associated ligament injuries. Plain X-rays were performed in 47 % of the patients and MRI scans in 86 % of the patients during the initial assessment of their injuries. Sixteen patients (11 %) were lost to follow-up 6–12 weeks after their surgery.

Mechanisms of injury included sports-related accidents in 58 % of the cases (football, rugby, cricket), falls (26 %), and road traffic accidents (5 %), while 5 % had no definite history of any specific trauma. Our patients belonged to three main categories of occupations: manual workers (28 %), office workers (23 %), and students (23 %), while 4 % of the patients were professional sportsmen. Indeed, most (79 %) of the patients included regular sporting activities in their daily routine (football, rugby, cricket, and gym exercises).

We used clinical symptomatic resolution as the outcome measure in our patients to assess the failure rate. Based on this assessment, 83 % (*n* = 113) of the meniscal repairs were assumed to have healed, as the patients had complete or significant resolution of their symptoms on subsequent regular follow-up. 17 % (*n* = 23) of the meniscal repairs were considered to have failed to heal due to ongoing or recurrent symptoms in these patients (pain, swelling, locking) (Table 1).

**Table 1:** Summary of outcomes for patients who underwent meniscal repair

	Number of meniscal repairs	Percentage success (%)	Failed repairs (%)	*p* Value
Age				
<25 years	83	87	13	0.80
>25 years	53	85	15	
Time of repair				
Early (<6 weeks)	82	91	9	0.49
Late (>6 weeks)	50	87	13	
ACL reconstruction				
With	81	86	14	0.20
Without (intact ACL)	53	84	16	
ACL reconstruction				
Simultaneous ACL	55	86	14	0.0006
Delayed ACL	26	77	27	
Zone of repair				
W/W	45	80	20	0.75
R/W	66	86	14	
R/R	12	84	16	
Side				
Medial	50	81	19	1.00
Lateral	86	88	12	

### Medial vs. lateral meniscal tears

63 % of the tears were present in medial and 37 % in lateral menisci. The failure rate was found to be 19 % in cases of medial and 12 % in cases of lateral meniscal repair (*p* = 1.00). 33 % of the tears (*n* = 45) were found to be present in the white-white zone, 48 % (*n* = 66) in the red-white zone, and 9 % (*n* = 12) in the red-red zone. The failure rate of meniscal repairs in the white-white zone was 20 % (*n* = 9), that in the red-white zone was 14 % (*n* = 9), and that in the red-red zone was 16 % (*n* = 2). Bucket handle tears comprised the majority of the tears (70 %), followed by transverse (5 %), radial (4 %), and longitudinal (3 %) tears.

### Associated ligament injuries

Ligament injuries were found in 83 patients (61 %) along with meniscal tears. These included acute ACL ruptures in 71 (52 %) patients, old ACL ruptures in 4 (3 %) patients, and recurrent ACL ruptures (which had been previously reconstructed) in 2 (1.5 %) patients. Six (4 %) patients had multiligament injuries. Of these 83 patients with ruptured ligaments, 55 (66 %) had simultaneous ACL reconstruction or reconstruction performed within 6 weeks of injury, while 26 (32 %) had their ACL reconstructed at a later stage following an initial meniscal repair (after 6 weeks of injury), and 2 (2.5 %) patients did not require reconstruction (no instability symptoms). Comparison of the results for the patients with an intact ACL with those for the patients with a reconstructed ACL (combined early and delayed) showed failure rates of meniscal repair of 16 % for the intact ACL group and 14 % for the reconstructed ACL group. In the reconstructed ACL group, further analysis revealed that patients who had ACL reconstruction performed early (at the same time as meniscal repair or within 6 weeks of injury) had a meniscal repair failure rate of 14.5 % (*n* = 12). In comparison, patients who had delayed ACL reconstruction (after an initial meniscal repair and after 6 weeks of injury) had a meniscal repair failure rate of 27 % (*n* = 22; *p* = 0.0006). The difference between these two groups was found to be statistically significant.

### Timing of surgery

60 % of the patients had meniscal repair surgery within 6 weeks after sustaining the injury (defined as “early repairs”) and 37 % had surgery more than 6 weeks after the injury due to their delayed presentation (defined as “late repairs”). The failure rate was found to be 9 % in early repairs and 13 % in late repairs (*p* = 0.49). This difference was not statistically significant.

### Young vs. old

In patients who were younger than 25 years (61 %), the failure rate was found to be 13 %, in comparison with a 15 % failure rate in patients who were older than 25 years (39 %; *p* = 0.80).

**Fig. 3:**
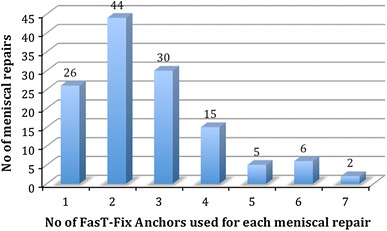
Locations of tears in different zones of menisci

No evidence of degenerative changes was seen in 79 % cases, while 19 % showed pre-existing grade I/II changes and 2 % showed grade III/IV changes in articular cartilage. Two patients (1.6 %) had postoperative complications, including 1 patient with tense hemarthrosis requiring further washout and 1 patient who developed DVT in the operated leg (calf) and was treated with warfarin. The number of FasT-Fix anchors ranged from 2 to 7 for each meniscal repair (mean 2.7) (Fig. 3).

### Failed meniscal repairs

The patients who were considered to have failed meniscal repairs (17 %, *n* = 23) underwent further investigations (MRI or CT arthrograms) and subsequently had repeat arthroscopy, which resulted in partial meniscal resection in 11 patients and re-repairs of the tears in 6 patients (the other 6 patients were lost to follow-up). The average age of the patients with failed repairs was 25.8 years (15–45 years). Another 10 % of the patients (*n* = 14) presented with recurrence of symptoms after initial resolution, with the recurrence occurring on average 5 months after surgery. Five of these patients had a history of recurrent trauma. Due to the persistence of their recurrent symptoms, after MRI or CT arthrograms, these patients underwent repeat arthroscopy which showed satisfactory healing of the menisci (complete or partial healing), without a new tear. On further follow-up, the symptoms in these patients gradually improved with physiotherapy within a few months.

## Discussion

No single accepted definition for failure of meniscal repair exists in the current literature. Noyes et al. [11] defined failure of the repair as the “persistence of symptoms (swelling, locking, or joint pain) and/or the requirement for repeat knee arthroscopy and meniscectomy.” There are three possible ways of identifying the healing (or failure) status of the repaired meniscus: repeat arthroscopy, repeat MRI scan, and correlation with clinical symptoms. Some studies have found that, on repeat arthroscopy after previous meniscal repair, the menisci were partially healed in the absence of ongoing clinical symptoms [12]. Muellner [13] showed that MRI does not have the ability to differentiate whether a meniscus has healed or not. Using clinical symptoms as a tool to assess the healing status provides only indirect evidence of successful healing. However, this is still to be accepted as an assessment tool because routine repeat arthroscopy in every patient to assess meniscal healing is not feasible in routine clinical practice. In addition, the patients may not want to be followed up once their symptoms have settled down after successful surgical management [11].

In young patients, sports-related injuries are usually the most common cause of a meniscal tear, accounting for more than one-third of all cases [14, 15]. The underlying mechanism of these injuries usually involves cutting or twisting movements and hyperextension [16]. Meniscal tears during these sports injuries have been reported to be accompanied by the rupture of the ACL in more than 80 % of cases [17]. In their study, Warren et al. [5] reported that the success rate of meniscal repair with ACL reconstruction can be up to 90 %, while the failure rate was 30–40 % when the knee remained unstable due to the ruptured ACL.

Our results showed that the failure rate was lower in cases of lateral meniscal repair. Previous studies have shown failure rates of 10 % for lateral and up to 40 % for medial meniscal repairs [18]. Our patients who had delayed ACL reconstruction had double the meniscal repair failure rate of the patients who had early ACL reconstruction along with meniscal repair (*p* = 0.0006). This is consistent with previous studies suggesting that 90 % of meniscal repairs are successful if the ACL is reconstructed at the same time as the meniscal repair, whereas failure rates of 30–40 % are seen if the knee remains unstable [5]. In addition to stability, ACL reconstruction is also considered to provide a favorable environment for meniscal repair healing due to intra-articular bleeding.

In our patients, the failure rate was only slightly better if meniscal tears were repaired within 6 weeks of injury. The available literature also does not suggest that the outcome changes depending on the age of the tear [19]. No major difference in the outcome was seen between different age groups of patients. The available literature does not suggest that the failure rate varies with patient age; however, in younger patients, meniscal preservation should be the preferred option in order to reduce the risk of subsequent arthritis, particularly for lateral meniscal tears. In our patients, the failure rate of meniscal repairs in the white-white zone was higher; this is consistent with previous studies which have suggested failure rates of up to 32 % in this zone [3].

Our study has a few limitations. It was a retrospective study. We used symptomatic resolution as the outcome measure in order to assess the success rate, and did not use objective scoring to accurately analyze our results. Our follow-up duration was short, and 11 % of the patients were lost to follow-up.

Our results have shown that the outcome of meniscal repair is statistically significantly better if ACL reconstruction is performed simultaneously with the meniscal repair (*p* = 0.0006). No significant dependence of the outcome on the age of the patient or the age or location of the tear was found (*p* > 0.05). However, considering the important role of the meniscus in maintaining knee function and preventing arthritis, meniscal preservation surgery should be considered whenever possible, especially in younger patients and cases of lateral meniscal tear.
